# First detection of human hepegivirus-1 (HHpgV-1) in Iranian patients with hemophilia

**DOI:** 10.1038/s41598-018-23490-4

**Published:** 2018-03-22

**Authors:** Yazdan Bijvand, Mohammad Reza Aghasadeghi, Fatemeh Sakhaee, Parviz Pakzad, Farzam Vaziri, Alireza Azizi Saraji, Fatemeh Rahimi Jamnani, Seyed Davar Siadat, Abolfazl Fateh

**Affiliations:** 1 Department of Microbiology, Faculty of Basic Sciences, North Tehran Branch, Islamic Azad University, Tehran, Iran; 2 Hepatitis and AIDS Department, Pasteur Institute of Iran, Tehran, Iran; 3 Department of Mycobacteriology and Pulmonary Research, Pasteur Institute of Iran, Tehran, Iran; 4 Microbiology Research Center (MRC), Pasteur Institute of Iran, Tehran, Iran; 5 Iranian Comprehensive Hemophilia Care Center, Tehran, Iran

## Abstract

A novel blood-borne virus called the human hepegivirus 1 (HHpgV-1) was recently discovered in hemophilia patients. The present study aimed to investigate the presence of HHpgV-1 in hemophilia patients. A total of 436 serum samples were investigated for the presence of hepatitis C virus (HCV), human pegivirus-1 (HPgV-1), torque teno virus (TTV), and HHpgV-1. Out of the 436 patients, 163 (37.4%), 19 (4.4%), 76 (17.4%), and four (0.9%) patients were positive for HCV, HPgV-1, TTV, and HHpgV-1, respectively. HHpgV-1 patients had a mean viral load of 4.9 ± 0.3 log RNA copies/mL and were co-infected with HCV-1a, HPgV-1, and TTV. Moreover, three HHpgV-1-positive patients exhibited stage F0 liver fibrosis. HCV viral load in HHpgV-1-positive patients was lower than those of HHpgV-1-negative patients. Results also revealed that co-infection of HHpgV-1 with HPgV-1 and HCV may play a protective role in patients with chronic HCV. In conclusion, we detected a low frequency of HHpgV-1 infection in hemophilia patients, and results suggested that HHpgV-1 infection was correlated with the presence of other blood-borne viruses and is likely to also correlate with low HCV viral load and reduced severity of liver disease. Additional studies are required to further investigate the clinical importance of HHpgV-1.

## Introduction

Blood-derived products from patients with hemophilia, treated by factor VIII concentrates, are possible sources of transfusion-transmitted infections (TTI). TTI, especially viral infections, such as viral hepatitis, or infections by human immunodeficiency virus (HIV) and human pegivirus-1 (HPgV-1; formerly known as GBV-C or HGV), can potentially be transmitted via blood transfusions^1^.

These infections were major health problems in the 1980s, when 65–75% of hemophilia patients were infected with HIV-1^2^.

This concern continued with the finding that approximately 80% of hemophilia patients treated with blood-derived products before 1992 were infected with hepatitis C virus (HCV)^3^.

Current screening practices and blood donor selection methods have improved TTI detection in blood-derived products, so that the residual risk of infection with hepatitis B virus (HBV), HCV, and HIV has decreased to approximately 1 or <1 million transfused units. However, despite this success, residual risk of infection remains^1,4^.

Kapoor *et al*. reported the discovery of human hepegivirus-1 (HHpgV-1), a new blood-borne virus that belongs to the *Flaviviridae* family and shares features with HCV and HPgV-1. HHpgV-1 was detected in serum specimens from two hemophilia patients and two blood transfusion recipients who received blood/plasma-derived products^5^.

Very little information is available on the prevalence of HHpgV-1. Accordingly, epidemiological studies from different parts of the world must be conducted to gain insight into the characteristics and disease mechanisms of HHpgV-1. Therefore, the present study aimed to investigate HHpgV-1, a new blood-borne virus, in Iranian hemophilia patients.

## Results

### Patient’s clinical characteristics

A total of 436 hemophilia patients were enrolled in this study. Of these patients, 288 (66.1%) and 148 (33.9%) were male and female, respectively. The mean age in all patients was 29.2 ± 8.3 years. In addition, mean ALT, AST, and ALK were 47.7 ± 29.9, 45.3 ± 25.4, and 175.2 ± 130.5, respectively (Table 1).

**Table 1:** Baseline and biochemical information between all hemophilia patients.

Variables	Hemophilia patients (n = 436)
Mean age ± SD	29.2 ± 8.3
Gender (male/female)	288/148 (66.1/33.9%)
ALT (IU/L) (mean ± SD)	47.7 ± 29.9
AST (IU/L) (mean ± SD)	45.3 ± 25.4
ALK (IU/L) (mean ± SD)	175.2 ± 130.5
HCV subtypes (n = 163) (%)	
1a	104 (63.8%)
3a	59 (36.2%)
Liver enzymes levels in HCV positive patients (n = 163)	
ALT (IU/L) (mean ± SD)	78.4 ± 11.6
AST (IU/L) (mean ± SD)	73.3 ± 12.2
ALK (IU/L) (mean ± SD)	276.1 ± 158.1
HCV viral load (IU/mL) (mean ± SD)	7.3 × 10^5^ ± 2.8 × 10^5^
HPgV-1 (%)	
Positive	19 (4.4%)
Negative	417 (95.6%)
HPgV-1 viral load (log RNA copies/mL) (mean ± SD)	5.2 ± 1.3
HHpgV-1 (%)	
Positive	4 (0.9%)
Negative	432 (99.1%)
HHpgV-1 viral load (log RNA copies/mL) (mean ± SD)	4.93 ± 0.34
TTV (%)	
Positive	76 (17.4%)
Negative	360 (82.6%)
Liver fibrosis stage (%)	
F0	44 (27.0%)
F1	85 (52.2%)
F2	24 (14.7%)
F3	10 (6.1%)
F4	0 (0.0%)

ALT, alanine aminotransferase; AST, aspartate aminotransferase; ALK, alkaline phosphatase; HPgV-1, Human pegivirus-1; HHpgV-1, Human hepegivirus-1; TTV, Torque teno virus; SD, standard deviation.

### Detection of HCV, HPgV-1 and TTV in hemophilia patients

The frequency of HCV, HPgV-1, and TTV in hemophilia patients was 163 (37.4%), 19 (4.4%), and 76 (17.4%), respectively. After genotyping for HCV, the frequency of HCV-1a was 104 (63.8%) and that of HCV-3a was 59 (36.2%). Liver fibrosis stages in chronic HCV infection were 44 (27.0%) in F0, 85 (52.2%) in F1, 24 (14.7%) in F2, and 10 (6.1%) in F3. The mean HCV and HPgV-1 viral loads were 7.3 × 10^5^ ± 2.8 × 10^5^ IU/mL (range, 1.2 × 10^5^–1.4 × 10^7^ IU/mL) and 5.4 ± 0.4 log RNA copies/mL (range, 4.8–6.0 log RNA copies/mL), respectively (Table 1).

### Frequency of HHpgV-1 and characterization of HHpgV-1 patients

We detected HHpgV-1 in four (0.9%) patients using semi-nested PCR assay and analyzed the isolates via partial- and whole-genome sequencing. Patients were named IHP-16, IHP-234, IHP-343, and IHP-381. HHpgV-1 patients were all male and had a median age of 66.3 ± 4.0 years old. In addition, these patients were simultaneously co-infected with HCV, HPgV-1, and TTV. The liver fibrosis stage of three patients was F0, while one patient was in the F1 stage. The mean viral load in all HHpgV-1-positive patients was 4.9 ± 0.3 log RNA copies/mL (range, 4.6–5.4 log RNA copies/mL), and is similar to those reported by Wang *et al*. and Coller *et al*.^11,13^. HCV viral load was significantly lower in patients with HHpgV-1 (*P* = 0.010). All patients had the HCV genotype 1a. Results revealed significant associations of HHpgV-1 infection with age (*P* = 0.001), HCV genotype (*P* = 0.002), HPgV-1 viral load (*P* = <0.001), TTV positivity (*P* = 0.001), and Liver fibrosis stage (*P* = 0.001). In addition, ALT (*P* = 0.542), AST (*P* = 0.547), and ALK (*P* = 0.118) levels were not associated with HHpgV-1 positivity. These results reveal that HHpgV-1 viremia may not affect liver enzymes levels in patients with HCV infection (Table 2).

**Table 2:** Properties and Baseline Clinical Characteristics of Iranian Patients based on HHpgV-1 positivity.

Variables	IHP-16	IHP-234	IHP-343	IHP-381	Total population (mean ± SD)	*P-*value
Sex	Male	Male	Male	Male	—	0.305
Age (years)	62.0	68.0	71.0	64.0	66.3 ± 4.0	0.001*
ALT (IU/L)	59.0	48.0	47.0	61.0	53.8 ± 7.3	0.542
AST (IU/L)	55.0	50.0	42.0	48.0	48.8 ± 5.4	0.547
ALK (IU/L)	350.0	342.0	384.0	280.0	339.0 ± 43.3	0.118
HCV genotype	HCV-1a	HCV-1a	HCV-1a	HCV-1a	—	0.002*
HCV viral load (IU/mL)	4.0 × 10^5^	3.5 × 10^5^	5.3 × 10^5^	4.3 × 10^5^	4.3 × 10^5^ ± 0.7 × 10^5^	0.010*
HPgV-1 viral load (log RNA copies/mL)	5.9	5.9	6.0	5.4	5.2 ± 1.3	<0.001*
TTV positivity	Yes	Yes	Yes	Yes	—	0.001*
HHpgV-1 viral load (log RNA copies/mL)	4.9	4.8	4.6	5.4	4.9 ± 0.3	<0.001*
Severity of fibrosis	F0	F0	F1	F0	—	0.001*

ALT, alanine aminotransferase; AST, aspartate aminotransferase; ALK, alkaline phosphatase; HPgV-1, Human pegivirus-1; HHpgV-1, Human hepegivirus-1; TTV, Torque teno virus; SD, standard deviation; *Statistically significant.

### Sequence and phylogenetic analysis of HHpgV-1

**Figure 1:**
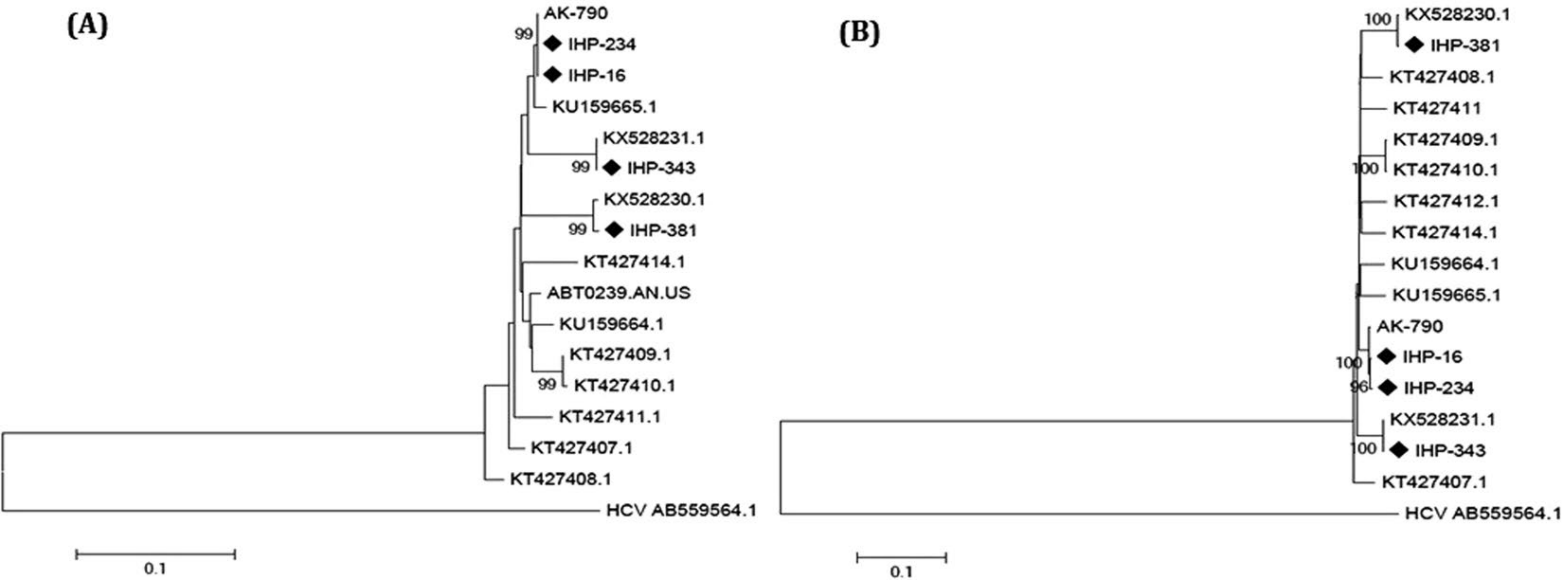
Phylogenetic tree based on a partial helicase gene sequence (**A**) and whole genome sequence of HHPgV-1 (**B**). Only bootstrap values >80% are shown.

Comparison of the partial genome sequences with previously published HHpgV-1 genomes showed that IHP-16 and IHP-234 shared 99% identity with KT439329.1 (AK-790), while IHP-343 and IHP-381 shared 99% identity with KX528231.1 and KX528230.1, respectively. Furthermore, comparison of the whole genome sequence with previously published HHpgV-1 genomes revealed that IHP-16 and IHP-234 showed 100% and 96% identity with KT439329.1 (AK-790) and IHP-343 and IHP-381 had 100% identity with KX528231.1 and KX528230.1, respectively (Fig. 1A,B).

## Discussion

This is a preliminary study to provide first evidence of the frequency of HHpgV-1 as new blood-borne virus in Iranian patients with hemophilia. Subsequent specific semi-nested PCR screening of hemophilia samples from 432 subjects failed to detect any HHpgV-1-positive samples. HHpgV-1 was also infrequently identified in four hemophilia patients, and subsequently, they were confirmed using Illumina sequencing. This result was in keeping with that of three previous studies, which detected low frequency of HHpgV-1 in hemophilia patients^5,14,15^.

In the current study, four positive samples had co-infection of HHpgV-1 with HCV, HPgV-1, and TTV. This result is completely consistent with data from four published reports, in which HHpgV-1 was mainly or only identified in serum containing HCV, HPgV-1 RNA, and other blood-borne virus. These results indicate the conclusion that HHpgV-1 circulates less frequently in humans but especially in patients with HCV infection is relatively frequent^5,13–15^.

Compared to HCV and HPgV-1, the low frequency of detection of HHpgV-1 in hemophilia patients as a high-risk group may be described by the fact that (I) HHpgV-1 is an uncommon viral infection in humans, (II) HHpgV-1 is less persistent, (III) due to the limited sample numbers and areas tested, or (IV) the PCR-based screening method was incapable to amplify sequences of more genetically divergent variants of HHpgV-1^5^. Whole-genome sequencing has been demonstrated to have high accuracy and sensitivity for the detection of new viral RNA, such as HHpgV-1 RNA, in human blood, which can be highly divergent and indiscernible using conventional PCR techniques^14,15^. One limitation of the present study is that not all samples were subjected to whole-genome sequencing because of limited funding.

After discovering HCV in 1989, HPgV-1 in 1995, TTV in 1997, TTV-like minivirus (TLMV), and SEN virus (SENV) in 2000, no more transfusion-associated virus infections have been reported^16^. In addition, many cases of post-transfusion hepatitis with unknown etiology still occur among blood-transfusion recipients. However, chronic HCV infection is a major health problem and leads to liver cirrhosis, hepatocellular cancer, liver transplantation, and death^17–21^. In return, it was reported that most HPgV-1 infections were usually asymptomatic, transient, and self-limiting^22^.

Interestingly, several studies have also shown that co-infection with HIV and HPgV-1 can delay progression to acquired immune deficiency syndrome (AIDS), which can be potentially explained by reduced viral replication and/or impairment in the host immune system^23–25^. Moreover, previous studies have revealed that co-infection with Ebola and HPgV-1 viruses was correlated with improved patient survival^26^.

In the current study, the mean HCV viral load in HHpgV-1-positive patients was lower than that in HHpgV-1-negative patients and three patients were in the F0 liver disease stage. Results suggested that co-infection of HCV with HHpgV-1 and HPgV-1 caused a reduction in HCV viral load and also decreased the severity of liver damage in chronic HCV. Berzsenyi *et al*. showed that HPgV-1 infection in chronic HCV was correlated with reduced liver disease severity^27^. Moreover, Kandathil *et al*. showed similar results that is, no evidence that HHpgV-1 leads to liver disease^15^. HHpgV-1, like HPgV-1, is presumably not a significant cofactor in the development of HCV-related liver disease. These results give us with clues about the medical consequences of HHpgV-1 infection, raising the eventuality that HHPgV-1 infection may lead in limited or absence of pathogenicity^13^. However, further studies are required to verify the mechanisms underlying the interactions of HCV with HHpgV-1 and HPgV-1.

In a very recent study, Wang *et al*. reported the tight association HHpgV-1 with HCV. Also, in line with our study, HHpgV-1 seems not to worsen HCV-related liver fibrosis. As a result, they showed that HHpgV-1 could effect on the replication and pathogenesis of HCV^13^.

Whether HHpgV-1 plays an important role in the development of human hepatitis remains unclear. HHpgV-1 has the characteristics of both hepaciviruses and pegiviruses, thereby increasing the risk of chronic infections like HCV and HPgV-1 infection. Given that the effects of HHpgV-1 on human health have not been well characterized, a more precise evaluation of HHpgV-1 frequency in human populations is warranted^28^.

HHpgV-1 is likely to be infectious and can potentially be added to the list of viral infections that are investigated at Hemotherapy Services; further experiments can be conducted to prove this issue^16^. Historically, the prevalence of HCV infection was underestimated and was resolved only when the full range of the genetic variation of HCV was characterized. However, continued efforts to recognize more cases of HHpgV-1 infection are ongoing, for better understanding of the epidemiology, pathogenesis, and clinical relevance of HHpgV-1^11^.

In conclusion, our results demonstrated that the low prevalence of HHpgV-1 infection in hemophilia patients may be correlated with ongoing HCV, HPgV-1, and TTV infection and may be associated with lower HCV viral load and reduced liver disease severity. However, additional studies are required to further characterize the clinical importance of HHpgV-1.

## Patients and Methods

### Study population

The current study was performed at the Pasteur Institute of Iran (PII) from October 2016 to August 2017 and conducted in accordance with the 1975 Declaration of Helsinki and relevant local regulations. The study was approved by the Ethical Committee of PII, and written informed consent was directly obtained from all participants. Serum samples from a total of 436 hemophilia patients were collected at the Iranian Comprehensive Hemophilia Care Center (ICHCC) and PII. Severity and staging of liver disease patients were extracted from the patient records. Patients were classified into the following five groups based on the severity of fibrosis: no fibrosis (F0), portal fibrosis alone (F1), portal fibrosis with rare septae (F2), portal fibrosis with many septae (F3), and cirrhosis (F4)^6^. Aspartate Aminotransferase (AST), alkaline phosphatase (ALK), and Alanine Aminotransferase (ALT) were detected according to laboratory protocols. All patients were negative for both hepatitis B surface antigen (Radim SPA, Pomezia-Rome, Italy) and HIV antibodies (Radim SPA, Pomezia-Rome, Italy).

### DNA and RNA extraction

Total RNA was extracted using the High Pure Viral RNA Kit (Roche Diagnostics Deutschland GmbH, Mannheim, Germany) and cDNA was synthesized using the Transcriptor First Strand cDNA Synthesis Kit (Roche Diagnostics Deutschland GmbH, Mannheim, Germany), according to the manufacturer’s instructions. TTV DNA was extracted using High Pure PCR Template Preparation Kit (Roche Diagnostics Deutschland GmbH, Mannheim, Germany), according to the manufacturer’s instructions.

### HCV detection and genotyping

A nested PCR assay was prepared for detection of HCV using the primer pair HCV-251F (5′-AGCGTCTAGCCATGGCGT-3′) and HCV-22R (5′-GCACGGTCTACGAGACCT-3′) during in the first round of PCR (264 bp) and HCV-FN-F2 (5′-GTGGTCTGCGGAACCGG-3′) and HCV-RN-R2 (5′-GGGCACTCGCAAGCACCC-3′) during the second round of PCR (174 bp). PCR conditions for the first round were 95 °C for 5 min, followed by 30 cycles of 94 °C for 45 s, 59 °C for 45 s, and 72 °C for 45 s, and 72 °C for 5 min as a final extension. PCR conditions for the second round were to the same as the first, but annealing temperature (62 °C) was different. PCR-restriction fragment length polymorphism assay (PCR-RFLP) was used to detect HCV genotypes. HCV-genotyping by RFLP assay has also been confirmed by sequencing of the 5′ non-coding region fragments^7^.

### HPgV-1 and Torque Teno Virus (TTV) detection

The primer pairs for detection of HPgV-1 were: HPg-F1 (5′-CCTTGGACCCAGGTICCNACIGA-3′) and HPg-R1 (5′-CCTGGTGGGGTRGCIGTNGC-3′) in the first round of PCR (242 bp) and HPg-F2 (5′-GGACCCAGGTGCCIACGGAYGC-3′) and HPg-R2 (5′-CCTGGTGGGGTRGCGGTNGCRTA-3′) in the second round of PCR (208 bp)^5^. The reaction involved an initial denaturation at 94 °C for 5 min, followed by 35 cycles of 94 °C for 45 s, 59 °C for 45 s, and 72 °C for 45 s, and a final extension at 72 °C for 7 min. Identical cycling conditions were applied during the second round, with an annealing temperature of 59 °C for the remaining 40 cycles.

Semi-nested PCR was used to detect the presence of TTV DNA. Three specific primers (NG059, NG061, and NG063) were used to amplify the viral DNA as previously described^8^. The 386 bp (first round) and 271 bp (second round) PCR products were analyzed by electrophoresis on a 2% agarose gel.

### Screening method of HHpgV-1

We used a semi-nested PCR method with helicase gene primers for the detection of HHpgV-1. The PCR primers HHpgV-ak1 (5′-GTTGTATTCGCCACAGCCAC-3′) and HHpgVak2 (5′-TCAAAGTTTCCTGTGTAGCCTGT-3′) were used in the first round and HHpgV-ak3 (5′-GTATTCGCCACAGCCACTCC-3′) and HHpgV-ak2 were used in the second round of semi-nested PCR^5^. The PCR conditions for the first round included 5 min of denaturation at 94 °C, followed by 35 cycles of 94 °C for 45 s, 57 °C for 45 min, and 72 °C for 45 min, and a final extension at 72 °C for 7 min. The PCR conditions for the second round were 5 min of denaturation at 94 °C, followed by 35 cycles of 94 °C for 45 s, 59 °C for 45 s, and 72 °C for 45 s, and a final extension at 72 °C for 10 min. The amplification products of the first and second PCR round were 317 bp and 314 bp, respectively.

### HCV, HPgV-1 and HHpgV-1 RNA quantification

HCV RNA viral load was measured using Amplicor Monitor HCV 2.0 (Roche Diagnostics Deutschland GmbH, Mannheim, Germany), according to the manufacturer’s instructions, respectively. A quantitative PCR (qPCR) method using primers and probes directed against the HHpgV-1 5′-untranslated region (UTR) and nonstructural proteins 2/3 (NS2/3) genes and 5′ UTR of the HPgV-1 was used to determine for the presence of HHpgV-1 and HPgV-1 viral RNAs^9–11^.

### Partial- and whole-genome sequencing and phylogenetic analysis

A partial helicase gene (nucleotide 4488 to 5021) of HHpgV-1 was sequenced. In brief, for purification of PCR products, we used the High Pure PCR Product Purification Kit (Roche Diagnostics Deutschland GmbH, Mannheim, Germany). Subsequently, purified PCR products were sequenced using an ABI Automated Sequencer (Applied Biosystems, Foster City, CA, USA).

All datasets were obtained using the Illumina HiSeq. 2500 platform (Illumina Inc., San Diego, CA, USA), yielding an average of 150 million reads per sequencing lane. All HHpgV-1 genome sequences in the NCBI GenBank Database were compared using MEGA6 using the neighbor-joining model and the maximum-likelihood algorithm, with 1000 bootstrap replicates applied to compute branch strength^12^. In addition, the outgroup sequence for the analysis was HCV (AB559564.1).

### Nucleotide sequence accession number

The HHpgV-1 complete genome has been submitted to GenBank under accession number MF775734, MG210577, MG210578, and MG210579 for IHP-16, IHP-234, IHP-343, and IHP-381, respectively.

### Statistical analysis

Statistical analyses were carried out using the IBM SPSS Statistics for Windows v.22.0 (IBM Corp. Armonk, New York). All statistical tests were two-tailed, and *P* < 0.05 was considered statistically significant. Fisher’s exact test/χ^2^ was performed to detect significant associations among qualitative variables, while the Mann-Whitney U-test was performed for continuous variables.
